# Celluloid

**Published:** 1880-09

**Authors:** 


### CELLULOID.

The display of celluloid at the last Paris Exhibition naturally drew much attention to this curious American product, although it had been manufactured in France for nearly three years, and was already quite extensively sold as " American coral." Much has since been published in reference to it, and one journal, the *Industrie Blatter*, of Berlin, took the trouble to send out a sample to each of its readers. Prof. Von Wagner, in his valuable *Jahresbericht*, gives a full account of it which we condense as follows:

Celluloid is made by dissolving pyroxyline (or guncotton) in camphor, instead of ether or alcohol. To prepare it for treatment with the camphor it is first ground in water. After the water has drained off, it is placed under pressure in a perforated vessel, and almost converted into a solid body, which, however, still contains enough misture to prevent spontaneous ignition in the subsequent operations. This mass is now intimately mixed with camphor by grinding them together in water. One part of camphor, by weight, is employed to two parts of pyroxyline, but other proportions can be employed with good results. The desired pigments and other substances are added along with the camphor. After they have all been very thoroughly mixed, the mass is subjected to a very heavy pressure, which removes all the moisture and also brings the camphor into more close contact with the pyroxyline to aid it in dissolving the latter. The dried and pressed mass is now put into a vessel of the form in which it is desired to have the celluloid. In the top of this vessel is a piston or plunger, so that it can be subjected to the action of a hydraulic press. While under pressure it is heated by steam or otherwise to from 140° up to 265° F., according to the quantity of the mixture. It is kept at this temperature and under this pressure until the camphor has dissolved all the pyroxyline. The temperature increases the solvent power, while the pressure keeps the ingredients in intimate contact. The result is a solid mass perfectly homogeneous throughout.

Artificial ivory is prepared from 100 parts of ivory dust, 100 of pyroxyline, and 50 of camphor. The pyroxyline is ground wet, then pressed until only enough water remains in it to prevent ignition. It is then mixed with the ivory dust and camphor, and pressed between absorbing cushions until all the moisture is extracted. Then 50 parts of nitrite of ethyl are added. The mixture is then left for several hours in a closed vessel until the nitrate is equally distributed throughout the mass. It is next subjected to heavy pressure in heated cylinders, as before described, and rolled between hot rollers. The product thus obtained has the appearance of natural ivory, is free from streaks and spots, is not attacked by moisture, and while hot can be pressed into any shape.

Celluloid as it leaves the press is about as dense as sole leather, but hardens in the air owing to a slight evaporation of camphor. In the finished product there is still a good deal of camphor, and herein is found the essential advantage in the use of camphor over ether, alcohol, and other liquid or volatile solvents. All such solvents are completely removed from the mass, while enough camphor remains in it perpetually to serve as solvent over and over again, and to give it the property of being readily changed into any other shape at a high temperature without the addition of any other solvent.

By another process a dilute solution of camphor is employed, 1 part camphor to 8 of alcohol, which will not dissolve pyroxyline at common temperatures but does so when heated. The pyroxyline is ground, mixed with pigment or dye, the water all removed, and 1 part of solvent added to 2 parts of pyroxyline, well stirred and put in a closed vessel until the solvent has saturated all parts of it. It is then heated under pressure as before described. The Compagnie Franco-Americaine, in Stains, near Paris, has been making celluloid for over three years, and has a branch at Mannhein, in Baden. The Rubber Comb Company in Hanover also took up its manufacture, but abandoned it again, owing, it is said, to the danger from fire. Renleaux is of the opinion that some experimenter should contrive a method for dispensing with the camphor, and also rendering the paroxyline less combustible; two difficult problems which Professor Wagner believes are not likely to be accomplished.

Unlike hard rbbber, celluloid does not become electrical when rubbed. The odor of camphor can only be noticed when the substance is warmed, or on being rubbed. The numerous uses to which it is applied are too well known to need repetition here.—*Boston Journal of Chemistry.*

